# Therapeutic potential and pharmacological significance of extracellular vesicles derived from traditional medicinal plants

**DOI:** 10.3389/fphar.2023.1272241

**Published:** 2023-12-01

**Authors:** Peijie Wu, Wenjun Wu, Shu Zhang, Jun Han, Chao Liu, Han Yu, Xiping Chen, Xiaofeng Chen

**Affiliations:** State Key Laboratory of Southwestern Chinese Medicine Resources, School of Basic Medical Sciences, Chengdu University of Traditional Chinese Medicine, Chengdu, China

**Keywords:** traditional medicinal plants, extracellular vesicles, lipids, pharmacology, therapy, tumor, inflammation

## Abstract

Medicinal plants are the primary sources for the discovery of novel medicines and the basis of ethnopharmacological research. While existing studies mainly focus on the chemical compounds, there is little research about the functions of other contents in medicinal plants. Extracellular vesicles (EVs) are functionally active, nanoscale, membrane-bound vesicles secreted by almost all eukaryotic cells. Intriguingly, plant-derived extracellular vesicles (PDEVs) also have been implicated to play an important role in therapeutic application. PDEVs were reported to have physical and chemical properties similar to mammalian EVs, which are rich in lipids, proteins, nucleic acids, and pharmacologically active compounds. Besides these properties, PDEVs also exhibit unique advantages, especially intrinsic bioactivity, high stability, and easy absorption. PDEVs were found to be transferred into recipient cells and significantly affect their biological process involved in many diseases, such as inflammation and tumors. PDEVs also could offer unique morphological and compositional characteristics as natural nanocarriers by innately shuttling bioactive lipids, RNA, proteins, and other pharmacologically active substances. In addition, PDEVs could effectively encapsulate hydrophobic and hydrophilic chemicals, remain stable, and cross stringent biological barriers. Thus, this study focuses on the pharmacological action and mechanisms of PDEVs in therapeutic applications. We also systemically deal with facets of PDEVs, ranging from their isolation to composition, biological functions, and biotherapeutic roles. Efforts are also made to elucidate recent advances in re-engineering PDEVs applied as stable, effective, and non-immunogenic therapeutic applications to meet the ever-stringent demands. Considering its unique advantages, these studies not only provide relevant scientific evidence on therapeutic applications but could also replenish and inherit precious cultural heritage.

## 1 Introduction

Extracellular vesicles (EVs), which are biological membrane-bound vesicles of 40–150 nm, are secreted by all mammalian cells (Colombo et al., 2014; Peng et al., 2020). Despite being initially considered as cellular debris and therefore underestimated, EVs are now recognized as significant factors in controlling numerous physiological and pathological mechanisms in multicellular organisms. Further investigation has revealed that these EVs are surrounded by a double layer of fats and proteins, containing various functional nucleic acids, lipids, proteins, RNAs, and other elements. Although most EVs share analogical structures, the uptake and signal transduction capacity primarily rely on the heterogeneity of their source and components. Due to these distinctive features, EVs play a vital role in facilitating intercellular communication and the transfer of information and exchange of materials in both prokaryotes and higher eukaryotes. Since their first discovery in the 1980s, mammalian extracellular vesicles (MEVs) have been extensively researched, and their biogenesis mechanisms and functions of mediating intercellular transmission have been gradually elucidated (Colombo et al., 2014; Abels and Breakefield, 2016; Isaac et al., 2021). For example, the arrangement of lipids in a bilayer enables MEVs to trap both water-loving and water-fearing molecular substances. Moreover, MEVs have also been reported to have remarkable specific targeting capabilities (Shao et al., 2020; Wu et al., 2022; Zhang et al., 2022). Intriguingly, EVs possess physiological activity and have the ability to cross bio-membrane barriers, evade phagocytosis by the reticuloendothelial system, and extend the lifespan of drugs (Duan et al., 2021; Nguyen and Fuhrmann, 2022; Xie et al., 2023). Although they exist in the nanodomain, EVs are considered low toxicity and capable of decomposing. As a result, extensive research has been conducted on EVs in diverse scientific fields, such as drug delivery systems, nutrition, disease treatment, and clinical diagnosis. Furthermore, EVs have proven effective in treating cardiovascular diseases, cancer, and neurodegenerative diseases and facilitating tissue repair (Cheng and Hill, 2022; Zhou et al., 2022). For instance, Qiao et al. developed a composite EVs based on HT1080 cells, which enclosed doxorubicin, leading to a substantial increase in the concentration of doxorubicin in the tumor tissue and augmenting its effectiveness against tumor cells (Qiao et al., 2020). In addition, MEVs are also applied to deliver vulnerable reagents, such as nucleic acid and lipids. According to recent research, it has been proved that the EVs obtained from dendritic cells contained siRNA, effectively reducing the expression of beta-site amyloid precursor protein cleaving enzyme-1 (Alvarez-Erviti et al., 2011). Moreover, EVs originating from brain endothelial cells have also been demonstrated to cross blood-brain bio-membrane barriers and transport the anti-tumor medications doxorubicin and paclitaxel to the brains of mice (Khan et al., 2021; Fang and Liu, 2022b). Based on these characteristics, the MEVs have been regarded as promising drug delivery strategies in previous studies.

**FIGURE 1:**
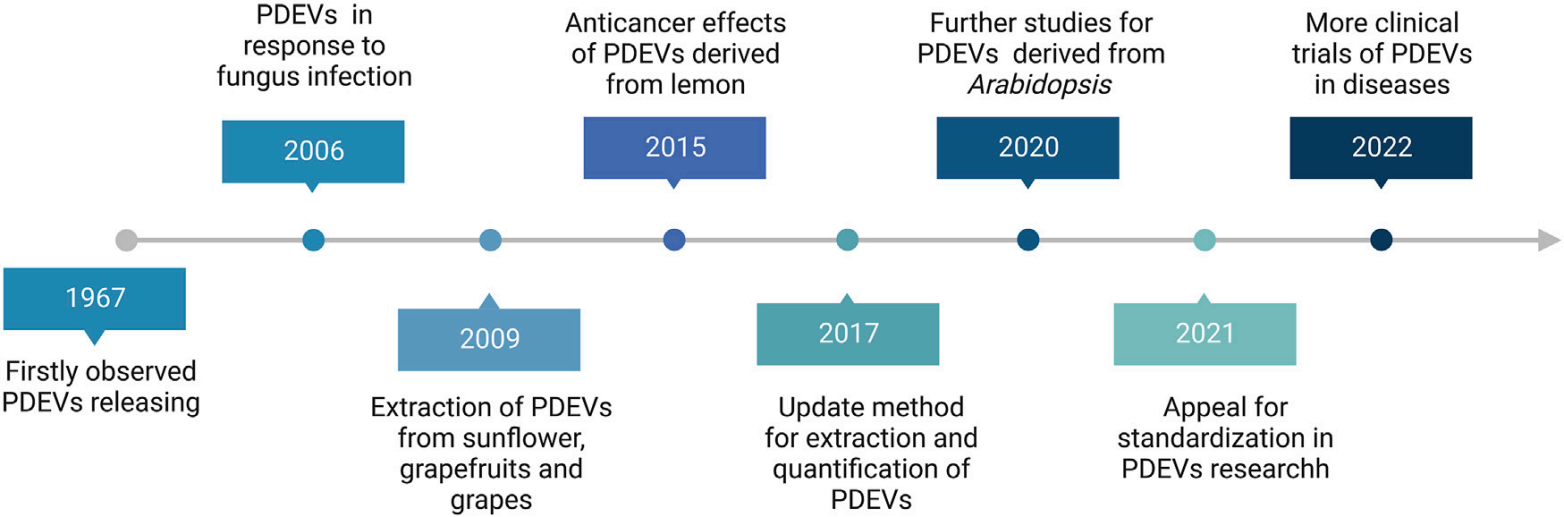
The brief research history of Plant-derived extracellular vesicles (PDEVs).

Despite the therapeutic potential of MEVs for drug delivery, their clinical application is still plagued by several challenges, including quick blood clearance, potential cancer stimulation, low production, and high expenses (Imai et al., 2015; Yamashita et al., 2016; Charoenviriyakul et al., 2017; Baldini et al., 2018). Accordingly, exploring safe, high-yield, and readily available sources of EVs has become a talking point for all researchers. According to the history of plant-derived extracellular vesicles (PDEVs), the first observed PDEVs were in 1967 and first isolated in 2009 (Figure 1). Since then, the research on PDEVs has been deepening and bringing the field into a period of diversified development. Interestingly, extracellular vesicles derived from plants (PDEVs) have been discovered in different plant species and possess exceptional qualities, making them a subject of increased interest in recent times. For example, PDEVs do not contain human or zoonotic pathogens and pose reduced immune risks *in vivo*. Hence, the PDEVs bestow non-immunogenic characteristics absent in MEVs and other artificial drug delivery methods (Zhuang et al., 2015; Baldini et al., 2018; Cao et al., 2019). PDEVs containing proteins, DNA, RNA, lipids, and other substances can acquire specific bioactive properties from the original plants, such as antioxidant, anti-inflammatory, and anti-cancer effects (Ju et al., 2013; Zhuang et al., 2015; Liu B. et al., 2020; Han et al., 2021). Furthermore, PDEVs, which have biological capabilities, can be utilized as carriers for molecules to enhance the therapeutic effects of drugs through synergistic effects. The transfer of these PDEV-loaded bioactive compounds can significantly impact the biological behaviors of recipient cells, particularly those related to various diseases like tumors and inflammation. Recent research has demonstrated that multiple PDEVs possess the ability to withstand challenging conditions in the gastrointestinal tract and overcome biological obstacles (Liu et al., 2023). It suggests that PDEVs may have great promise as platforms for delivering drugs. Various separate teams have utilized biochemical technologies to show the significant functions of PDEVs as potential intermediaries in intercellular communication and the exchange of bio-information among different species (Feng et al., 2023). For instance, it has been demonstrated that *Helianthus annuu* derived PDEVs containing lectins play a role in inflammation, cancer, and autoimmunity disease (Regente et al., 2009). Phosphoric acid can be transported by PDEVs derived from *Zingiber officinale Roscoe* to enhance chronic periodontitis while regulating pro-inflammatory cytokines can help alleviate colitis symptoms and preventing microglia activation can moderate encephalitis (Sundaram et al., 2019; Liu and Liu, 2022; Sundaram et al., 2022). Furthermore, various PDEVs from *Aloe ferox*, *Acrotome angustifolia*, Beta vulgaris, *Anthemis arvensis*, and *Vaccinium myrsinites* are being developed for disease therapy (De Robertis et al., 2020; Zeng et al., 2021; Chen et al., 2022; Mahdipour, 2022). Although growing evidence has also revealed that PDEVs could be absorbed by animal cells, some differences still exist between PDEVs and MEVs. For example, an apparent difference between PDEVs and MEVs is that PDEVs are abundant in phospholipids, such as phosphatidylethanolamines (PE), phosphatidic acids (PA) and phosphatidylcholine (PC). In contrast, MEVs mainly comprise sphingomyelins and cholesterol (Zhang et al., 2016b). Here, we summarized the advantages and disadvantages of PDEVs and MEVs in Table 1.

**TABLE 1:** The advantages and disadvantages of PDEVs and MEVs.

Strategies	Advantages	Disadvantages	References
PDEVs	Diverse types and abundant sources; Low toxicity; Relatively high extraction efficiency; Excellent drug carriers; High in phospholipids, easily absorbed; Excellent oxidation resistance; Broad adaptability to inflammatory diseases; Improve gut microorganisms	The database is incomplete, and the identification of specific functions and components is difficult; High dose dependence; There are few clinical trials, and the reliability of research results is low	Logozzi et al. (2022), Qin et al. (2022), Ratnadewi et al. (2022), Shinge et al. (2022), Wei et al. (2023)
MEVs	High stability; High stability in the digestive system; Less adverse reactions; Great potential for artificial transformation	High cost	Suharta et al. (2021), Cong et al. (2022), Liu et al. (2022), Wei et al. (2023)
		The database is incomplete, and the identification of specific functions and components is difficult; High dose dependence; There are few clinical trials, and the reliability of research results is low	

Accordingly, the magnitude of PDEVs in the field of biomedicine has witnessed a growing appreciation. Although PDEVs were discovered approximately a decade before MEVs, there is still limited understanding of the research conducted on drug delivery systems associated with PDEVs (Duan et al., 2021; Xu et al., 2021; Nguyen and Fuhrmann, 2022; Zhou et al., 2022). Therefore, this review aims to offer a brief summary of the PDEVs investigation, including the generation, formulation, thorough analysis, and loading methods in detail. Specifically, the focus is on revealing the capacity of PDEVs as therapies for diseases and as effective systems for delivering drugs based on ongoing research. We also tried to thoroughly evaluate the role of PDEVs as a lucrative mechanism for delivering drugs. In conclusion, we present our distinct perspectives on pharmacological significance, toxicity and immunogenicity, clinical utilization, possibilities, and obstacles of PDEVs.

## 2 Biogenesis of PDEVs

The presence of cell walls implies that plant cells possess more intricate communication methods than MEVs, making it difficult to investigate the forming process of PDEVs. According to recent research, the potential development of PDEVs may occur in various ways, such as through multivesicular bodies (MVBs), exocyst-positive-organelles (EXPOs), autophagosomes, and vacuolar pathways (Cui et al., 2020; Fang and Liu, 2022a). The primary types of PDEVs consist of the endosome (50–150 nm), microvesicles generated by the protrusion of the cell membrane (150–1,000 nm), and apoptotic bodies (1–1.5 μm), categorized by their origin within the cell, biological purpose, and physical characteristics (Fang and Liu, 2022a; Cheng and Hill, 2022).

The combination of MVBs and the plasma membrane (PM) results in the liberation of the intraluminal vesicle (ILV) and is controlled by the endosomal sorting complex required for transport (ESCRT), which is the predominant route for EVs creation in mammals. MVB pathway in plant cells bears a resemblance to that of mammalian cells (van Niel et al., 2022). The first observation of the MVBs occurred in the 1960s through a transmission electron microscope (TEM) (Ly et al., 2023). Numerous studies on different subjects, including unconventional protein secretion (UPS) and interactions between plants and pathogens, have validated the presence of PDEVs and their biogenesis mechanisms (An et al., 2006; Regente et al., 2012). For example, MVBs could discharge vesicles by merging with the PM or releasing them to particular infection locations while the fungus invades plant tissue (An et al., 2006). Multiple studies have additionally indicated that these MVBs, originating from the leaf tissues of *Arabidopsis thaliana* at the site of fungal infection, could be marked with Rab5-like GTPase ARA-6 and TETRASPANIN(TET)8-GFP (Cai et al., 2018; Cai et al., 2019). Specifically, the ARA-6 is situated at the site of infection and persists on the PM for subsequent transportation. Simultaneously, the Tetraspanin-8 is located on the released vesicles, resembling the CD63 marker of MEVs, and is anticipated to function as a marker for plant EVs (Cai et al., 2018). In addition, studies also proved that the fusion between MVBs and PM was also promoted by plant-microbe symbiosis, and these EVs were released into the periarbuscular space between plants and fungi.

There is also supporting evidence for alternative routes, including secretion through EXPO, autophagosomes, and fusion between vacuoles and the plasma membrane (Ly et al., 2023). Autophagosomes and EXPO are spherical double-membrane organelles. In spite of their comparable structure, EXPOs do not coexist with autophagosomes, nor do they experience any impact from nutrient deprivation. As a result, these two cellular components are regarded as separate entities. EXPO, possessing a dual-layered composition, has the capability to merge with the plasma membrane and discharge its internal vesicles into the extracellular space (Wang et al., 2010). However, EXPO exhibits morphological differences compared to MVBs and operates separately from endosomes and autophagosomes. Following fusion with the PM, EXPO secrete vesicles into the extracellular environment, which are larger by 200–500 nm (Cai et al., 2021). The recent discovery that glycosyltransferases and EXPO markers are found together in tobacco indicates that PDEVs derived from EXPO might play a role in releasing arabinogalactan proteins in plants. In addition to the MVBs and EXPO pathways discussed earlier, vacuoles may also play a role in forming PDEVs. Vacuoles containing hydrolytic enzymes are crucial organelles in plant defense (Pérez-Bermúdez et al., 2017; Wang et al., 2017). To combat pathogen infection, plants regulate the merging of vacuoles and plasma membranes, releasing substances like hydrolases and defense proteins into the external environment (Hatsugai et al., 2009). According to recent research, it has been discovered that central vacuoles in Arabidopsis originated from the transition of MVBs to small vacuoles (SVs) and the subsequent fusion of SVs (Cui et al., 2019). The fusion of autophagosomes and MVBs in yeast cells results in the formation of secondary vesicles, indicating a potential co-secretion of PDEVs through these secretory pathways (Manjithaya and Subramani, 2010).

Consequently, PDEVs have been discovered to have a crucial role in protecting the immune system and facilitating communication between cells. Nevertheless, the precise mechanisms of biogenesis for PDEVs remain uncertain, and PDEVs from various plant origins might be released in intricate ways. Hence, it is imperative to conduct additional research on the biogenesis process of PDEVs, as this could potentially aid in the systematic regulation of their secretion in the future.

## 3 Preparation and characterization of PDEVs

Utilizing PDEVs as naturally sustainable raw resources could be a promising approach in the medical field (Hernández-Díaz et al., 2021). MEVs have demonstrated significant promise in the biomedical domain, and PDEVs have also garnered considerable interest. In comparison to the MEVs, PDEVs offer a more comprehensive range of choices and enhanced safety (De Palma et al., 2020; Ly et al., 2023). However, achieving successful isolation of these PDEVs may be essential for practical applications. PDEVs are typically obtained directly from the extracellular surroundings, like apoplastic fluids or root exudate. These involve immersing plant raw samples in an infiltration buffer and isolating PDEVs by multiple methods, including ultracentrifugation, precipitation with polyethylene glycol, immunoaffinity, and size exclusion chromatography (Wang et al., 2021; Ali et al., 2022; Lai et al., 2022; Yin et al., 2022). Actually, the proper separation technique should be applied according to the final purpose of the PDEVs, among which basic research and clinical application are the bases for the selection of methods (Ströhle et al., 2022). However, there are currently limited separation methods for PDEVs, most of which refer to MEVs (Jackson et al., 2023). Due to the differences between animal cells and plant cells, such as starch and cellulose, the same isolation technique may be less effective in the extraction of PDEVs (Gardiner et al., 2016). As a result, we summarized the advantages and disadvantages of different isolation methods in Table 2 and provided a brief overview of the typical preparation and characterization of PDEV. It is predictable that an appropriate separation method could be more conducive to the acquisition of PDEVs with high purity, high yield, and high activity.

**TABLE 2:** Typical strategies and examples of applications to prepare PDEVs.

Strategies	Isolation condition	Advantage	Disadvantage	References
Ultracentrifugation	A range of centrifugation with different speeds and gradient ultracentrifugation. Considered as the gold standard in EVs extraction because of its capacity to extract EVs in a relatively high-purity fashion	Fast procedure; no limitations on sample volume; pure preparations; low cost	Vesicles trapping; loss of PDEVs; the possibility of clogging	Zhang et al. (2021)
Precipitation	Combination of centrifugation and clustering agents like PEG 6000 to trap EVs	The simplicity of the procedure, low cost, preservation of PDEVs integrity	Retention and contamination of the polymer	Jia et al. (2022)
Immunoaffinity	Using the interaction between the antigens on the surface of EVs and matching antibodies	Purity, high selectivity; convenient operation, low cost	High reagent cost; low yields; difficult to analyze complete vesicles	Sundaram et al. (2020)
Size-based isolation (SEC)	Separating similar-sized particles with ultrafiltration, size-exclusion chromatography, and flow field-flow fractionation, which typically resulted only in an EVs-enriched sample	Purity; reproducibility; preserves vesicle integrity; scalability; prevents PDEVs aggregation	Complexity; limitations on sample volume; specialized equipment; a small number of samples; high cost	Sidhom et al. (2020b)
Commercial isolation kit	EVs isolation kit	Suitable for small samples, simple steps, fast procedure	Impurity; low production; expensive reagents	Muraoka et al. (2020)

### 3.1 Preparation of PDEVs

The preparation of PDEVs follows similar methods established for MEVs. The extraction and purification technique used could be vital for the functioning of PDEVs since they are frequently present in a highly intricate environment. In existing research, numerous studies have aimed to enhance both extraction specificity and efficiency (Yang et al., 2021). Every method has its unique benefits and limitations. For example, a more precise strategy, like immunoaffinity, usually yields a pure sample but lacks efficiency and recovery. On the other hand, several methods, like membrane filtration, which have high efficiencies, may lack specificity and purity. However, it is important to mention that the functioning of PDEVs relies on the undamaged bilayer membrane (Ju et al., 2013; Wen et al., 2023). Therefore, it is crucial to exercise caution when extracting and storing it to avoid any deterioration. It can be achieved by employing protease inhibitors, keeping them at low temperatures, avoiding the freeze-thaw process, and maintaining a neutral pH (Li et al., 2017; Cheng et al., 2019). Extraction methods such as ultracentrifugation, ultrafiltration, immunoaffinity capture, precipitation, or microfluidics often rely on their distinct properties, such as their density, size, shape, and protein composition (Suharta et al., 2021). For instance, some studies suggested that blending is a superior choice for acquiring increased amounts of phytochemicals from grapefruits compared to juicing or manually squeezing (Uckoo et al., 2012). Wang and others determined that out of blending, high-speed centrifugal juicing, and low-speed juicing, the latter two preserved the most excellent phytochemical profiles and antioxidant activities of 19 vegetables, such as *Brassica oleracea*, *Raphanus raphanistrum*, and *Beta vulgaris* (Wang et al., 2021). This section summarized the typical techniques frequently employed for isolating PDEVs (Table 2).

#### 3.1.1 Ultracentrifugation

**FIGURE 2:**
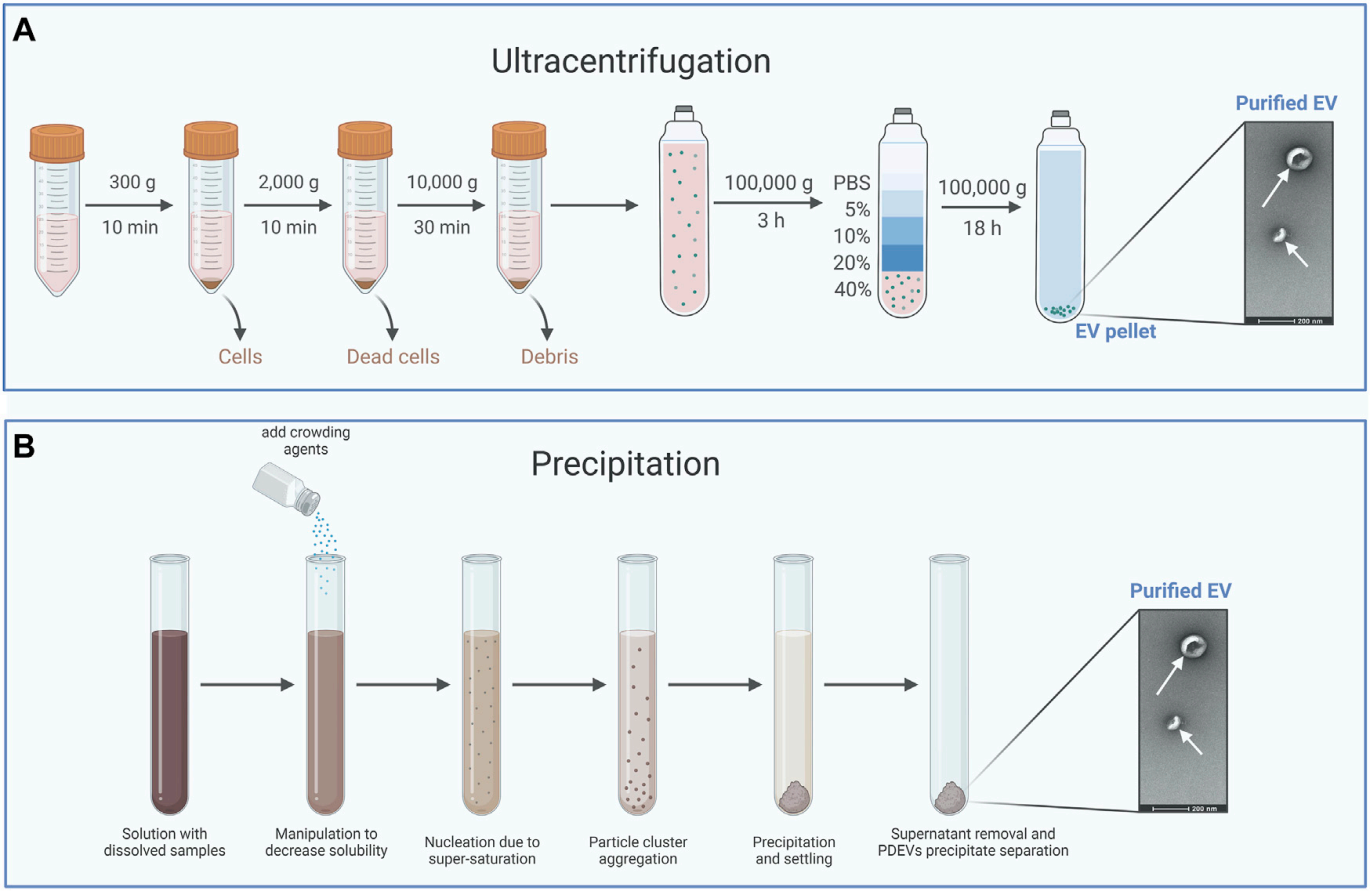
The typical method for the isolation of PDEVs. **(A)** Ultracentrifugation is often utilized as the primary method for extracellular vesicle isolation and usually divides into a differential or a density gradient centrifugation. **(B)** Precipitation isolates PDEVs by regulating the solubility and dispersion of PDEVs. For the separation of PDEVs, polyethylene glycol (PEG) is commonly employed as a coprecipitate.

Among the techniques mentioned above, ultracentrifugation is often utilized as the primary method for EVs isolation due to its straightforwardness, user-friendliness, cost-effectiveness over time, reasonable time requirement, and lack of complex sample preparation (Li et al., 2017). Accordingly, ultracentrifugation usually divides into a differential or a density gradient centrifugation (Figure 2A). Differential centrifugation relies on the separation of particles by their density, size, and shape through a sequence of high-speed centrifugation in a uniform medium (Li et al., 2017; Qiao et al., 2020). Actually, PDEVs are commonly extracted using the method of differential centrifugation (Gardiner et al., 2016). Various sizes of particles in the medium can separate different components with different rates of sedimentation. Generally, the intact cells could be eliminated at a low speed through centrifugation. At the same time, high-speed centrifugation can separate the sizable pieces and undamaged organelles. Ultimately, PDEVs are separated and enhanced through ultracentrifugation. It should be emphasized that prolonged and repeated ultracentrifugation can potentially disrupt the structure of the PDEVs. However, an additional cushion was incorporated at the base of the centrifuge tube, demonstrating its efficacy in enhancing the condition (Li et al., 2017; Yang D. et al., 2020; Lai et al., 2022). Differential centrifugation is the most common technology to extract PDEVs now. Despite the drawbacks of time-consuming processes and expensive costs, this technology offers the benefits of easy operation and a plentiful yield of PDEVs.

The density gradient centrifugation principle is based on the varying sedimentation coefficients of particles. Different particles settle in the area with varying density gradients under the influence of centrifugal force (Lai et al., 2022). Sucrose density gradient centrifugation is commonly used for purifying PDEVs that are acquired through differential centrifugation. The suspension of PDEVs was included in the solution with sucrose gradients. Following ultracentrifugation, the majority of PDEVs were typically concentrated within the density range of 1.13–1.19 g/mL. The primary origin of PDEVs is the sucrose gradient found in the sucrose layer, ranging from 30% to 45% (Stanly et al., 2019; Yang D. et al., 2020). Density gradient centrifugation yields PDEVs with excellent purity and a well-maintained structure, although the process is intricate and time-intensive. Accordingly, the most mature technique for preparing PDEVs is the combination of density gradient centrifugation and differential centrifugation.

#### 3.1.2 Precipitation

By regulating the solubility and dispersion of PDEVs, it is possible to induce the precipitation of PDEVs from the solution (Figure 2B). For the separation of PDEVs, polyethylene glycol (PEG) is commonly employed as a coprecipitate (Fang and Liu, 2022b). Hence, a less expensive technique for extracting EVs was developed, involving a sequence of centrifugation steps and purification with polyethylene glycol-6000 (PEG6000). The presence of this overcrowding agent has the potential to form a mesh-like arrangement capable of ensnaring PDEVs prior to their precipitation (Ali et al., 2022). The utilization of this technique can be extended to cause the precipitation of tiny particles and even viruses (Adams, 1973; Lewis and Metcalf, 1988). By modifying the PEG-6000 concentration employed during purification, this technique successfully yielded diverse average nanoparticles, measuring 365 nm, 304 nm, 256 nm, and 252 nm at PEG-6000 concentrations of 8%, 10%, 12%, and 15%, respectively, in contrast to the 403 nm achieved through ultracentrifugation. Despite having a lower yield and reduced bioactive polyphenol compounds, the PDEVs acquired through this technique exhibited comparable efficiency in macrophage cell uptake and profiles of small RNAs, proteins, and lipids compared to those obtained through ultracentrifugation (Kalarikkal et al., 2020). In addition, the precipitation technique tends to co-precipitate additional impurities that are not EVs, such as different vesicles, aggregates, protein complexes, and proteins of significant size (Zarovni et al., 2015; Castillo et al., 2018). For instance, Sreeram and others designed a technique for purifying PDEVs extracted from ginger roots utilizing PEG6000. These PDEVs precipitated by PEG6000 possess identical dimensions, capabilities, and biochemical makeup to PDEVs obtained through ultracentrifugation (Kalarikkal et al., 2020). Due to the straightforward procedure and excellent productivity, these techniques have also been employed in extraction kits (Umezu et al., 2021). While coprecipitation methods to isolate PDEVs exclusively have been limited to a few researchers, resulting from the tendency of coprecipitation techniques to cause EVs aggregation and coprecipitation of contaminants like lipoproteins (Gardiner et al., 2016).

#### 3.1.3 Immunoaffinity

**FIGURE 3:**
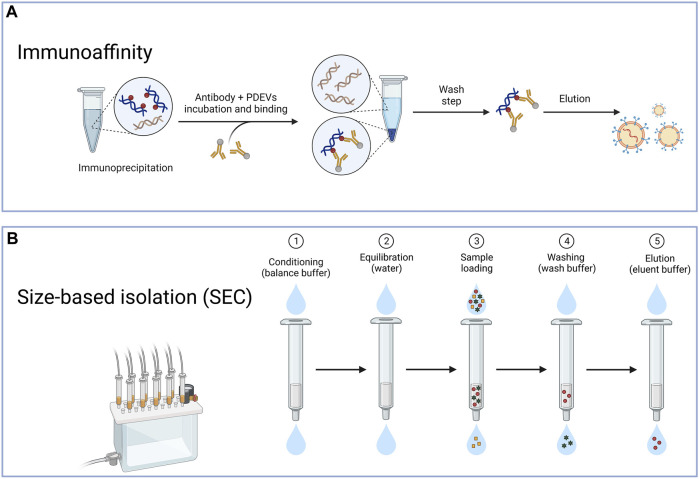
The typical method for the isolation of PDEVs. **(A)** The immunoaffinity technique is also frequently used to extract PDEV based on the interactions between identifying particular antibodies and distinct proteins located on the surface of extracellular vesicles; **(B)** Another method for preparing PDEVs is based on size exclusion chromatography (SEC) through the correlation between the hydrodynamic radius of the particles present in the sample and the pore size of the resin beads within the column.

The immunoaffinity technique is also frequently used to extract PDEVs (Figure 3A). The success of this method relies on the interactions between identifying particular antibodies and distinct proteins located on the surface of EVs. The protein target must be exclusively present or abundant within the EVs, bound within the membrane, and not present as a soluble component in the sample (Zarovni et al., 2015). The immunoaffinity technique can coat magnetic beads with specific antibodies that target proteins found in the membrane of PDEVs (Gardiner et al., 2016). This technique exhibits a high level of specificity and rapid separation rate. For instance, recent research successfully utilized the interaction between the phosphatidylserine on the surface of EVs and the T-cell immunoglobulin and mucin domain-containing protein 4 (Tim4) based on Ca2^+^. Therefore, it allowed the liberation of the EVs from the beads through the application of Ca2^+^ chelators (Yang M. et al., 2020). Furthermore, Field-flow fractionation (AF4) is an innovative method of isolating PDEVs based on their particle dimensions (Sitar et al., 2015). The Field-flow fractionation, in combination with light scattering detection, is appropriate for separating and characterizing a diverse range of analyses due to the vast array of mobile eluents available. For example, the AF4 method effectively isolated EVs from B16F10 cells (Zhang et al., 2018). Furthermore, PDEVs were effectively isolated from citrus and bitter gourd through dialysis and electrophoresis. The morphology and quantity of PDEVs separated using this technique were comparable to ultracentrifugation, as revealed by nanoparticle tracking analysis (NTA) or transmission electron microscopy (TEM) analysis (Yang M. et al., 2020; Yang et al., 2021). Additionally, aqueous biphasic systems were employed to extract PDEVs from garlic, with consistent particle size and a diverse range of labeled proteins (Özkan et al., 2021). However, the immunoaffinity technique has a limitation due to the heterogeneity of PDEVs. And only a fraction of EVs in a sample would contain the desired antigen. In contrast, the remaining subpopulation lacking this antigen would not be trapped by the antibody employed in the laboratory. Consequently, resulting from the absent study for the surface composition of PDEVs, the immunoaffinity method has not been studied extensively for extraction.

#### 3.1.4 The size exclusion chromatography (SEC)

Another method for preparing PDEVs is based on size exclusion chromatography (SEC) (Figure 3B). By considering the correlation between the hydrodynamic radius of the particles present in the sample and the pore size of the resin beads within the column, this technique also effectively separates EVs (Hajj et al., 2013; Fang and Liu, 2022b). Due to the larger hydrodynamic radii, EVs were unable to pass through the pores, leading to their early elution. In contrast, components with smaller hydrodynamic radii, capable of entering the pores, experienced late elution. Some studies evaluated the efficacy of ultracentrifugation, precipitation with polyethylene glycol, and SEC for isolating PDEVs from cabbage. The SEC technique was employed to isolate PDEVs, which exhibited greater yields and more comprehensive functional properties compared to ultracentrifugation (Monguió-Tortajada et al., 2019; Martínez-Greene et al., 2021). In regards to purity, SEC produced outcomes that were over 20 times greater than the two techniques mentioned before (Nordin et al., 2015). These EVs also could preserve biological functionality and structure intact. Jae et al. successfully applied SEC to isolate PDEVs from carrots and cabbage, resulting from the SEC not exerting more changes in their physicochemical properties (You et al., 2021). The SEC method is recognized as the primary technique for extracting PDEVs due to its simplicity, scalability, affordability, and rapid separation rate. It has been applied to extract PDEVs from cucumbers, tomatoes, and peppers (Sidhom et al., 2020a; You et al., 2021). As SEC depends on gravity, it has the potential to maintain the original bioactivity and structures of PDEVs. Although obtaining PDEVs with higher purity can also be achieved by utilizing HPLC, challenges may arise in terms of scaling up and the availability of equipment (Yang D. et al., 2020).

### 3.2 Characterization and contents of PDEVs

As the most potential candidate for drug delivery platforms, biochemical and physical characterization of PDEVs may be crucial. For example, accurate analysis of their physicochemical properties is significant for drug delivery applications. The description of the characterization and structure of particles also can verify the effective extraction of PDEVs. PDEVs have been reported to transfer multiple cargoes to recipient cells and transform their phenotype, containing the surface morphology, lipids, proteins, RNA, and small molecular substances of PDEVs (Wang et al., 2022). For instance, PDEVs packaged with secondary metabolites could facilitate them to pass the membrane barrier (Woith et al., 2021). Accordingly, these bioavailable molecules encapsulated on PDEVs make them unique and powerful tools for health-beneficial purposes. However, an extensive database of the physicochemical properties related to PDEVs is still unavailable due to the diversity of plants. Therefore, we summarized the currently typical characterization of PDEVs to display their natural clinical and pharmaceutical benefits.

#### 3.2.1 Morphological and physicochemical characterization

The assessment of morphology is essential in the study of PDEVs since it offers researchers direct insights into the characteristics. In most studies, the features, including shape, size, and surface charge, have been the primary focus of physical investigations of PDEVs. For instance, the majority of studies have adopted TEM and revealed that the morphology of PDEVs is typically round or saucer-shaped and characterized by lipid bilayers with lipid bilayers, which is regarded as the classic extracellular vesicle morphology. The size distributions of PDEVs separated through the disruption process range from 18–1,000 nm and exhibit negative surface charges. Recent studies suggested that the alteration of environmental pH or temperature may modify the size and charge of PDEVs. Up to now, PDEVs have been characterized by several approaches, including dynamic light scattering (DLS), nanoparticle tracking analysis (NTA), and electron microscopy (EM). Dynamic light scattering (DLS) has the capability to evaluate the hydrodynamic size of PDEVs through the fluctuation of light scattering. However, the size of PDEVs may not be accurately analyzed by dynamic light scattering, resulting from their polydispersity and potentially low scattering contrast (Sokolova et al., 2011; Hadisurya et al., 2023). The size may differ depending on the testing technique, as each method operates based on distinct principles and preparations. Moreover, nanoparticle tracking analysis (NTA) is the most common method to assess the size distribution and provides information on the number of PDEVs (McIlvenna et al., 2023). Despite the absence of standardized configurations and the limited scattering index of PDEVs, NTA is still the prevailing method for characterizing PDEVs.

In addition, the stability and optimal preservation of PDEVs have not been extensively elucidated compared with MEVs or synthetic liposomes. However, it is crucial to clarify the capability of PDEVs for biological management and scalability through these necessary measures. Therefore, various experiments have been carried out to examine the durability of PDEVs in diverse environments by modifying variables such as pH ranges, temperature, freeze-thaw cycles, and external manipulation. For example, numerous studies implicated that PDEVs derived from *Z. officinale*, *Daucus carota*, and *Aframomum sulcatum* could remain stable across a broad pH range under physiological temperatures (Mu et al., 2014). Moreover, the pH level may affect the size and surface charge of PDEVs. For example, PDEVs derived from *Citrus paradisi* maintained their size and surface charge in neutral and alkaline pH conditions while increasing in acidic conditions (Wang et al., 2014). Similarly, the PDEVs of *Z. officinale* exhibited an increased size and a transition in surface charge from negative to positive in an acidic solution (Zhuang et al., 2015; Zhang et al., 2016a). PDEVs derived from *Citrus paradisi* and *Citrus limon* also showed significant resistance to the digestive environment in both simulated conditions and mouse models. In addition, several studies indicated that PDEVs might be unstable under boiling or sonication. The antiproliferative function of PDEVs extracted from citrus fruits was destroyed under sonication or boiling conditions (Alzahrani et al., 2023). Consistently, the PDEVs obtained from apples were susceptible after boiling and sonication (Fujita et al., 2018). PDEVs derived from *Ginseng quinquefolium* could promote the polarization of macrophages compared with un-sonicated samples (Cao et al., 2019). It is crucial for researchers to understand that sonication is a commonly employed technique for drug incorporation (Wang et al., 2013; Zhang et al., 2016c; Zhuang et al., 2016; Ly et al., 2023; Raguraman et al., 2023). Therefore, the morphology of PDEVs should be evaluated during drug loading. PDEVs have been extensively applied as drug carriers, resulting from their adjustable dimensions and excellent stability. For example, it is possible to improve the stability and bioavailability of the delivery platform by combining hydrophobic agents with *Z. officinale* isolated from PDEVs. Without affecting biological activities, *Citrus paradisi*-derived PDEVs could be modified with biomolecules such as zymosan A, folic acid, or curcumin. Moreover, these lipid particles exhibited more excellent stability compared to cationic liposomes when exposed to a 10% bovine serum solution at 37°C (Wang et al., 2013).

#### 3.2.2 Proteins

The description of the distinctive protein composition and function in PDEVs is crucial for further classification of PDEVs. According to the International Society for Extracellular Vesicles (ISEV), it is recommended that researchers should document a minimum of three proteins in each EVs sample, such as transmembrane and cytosolic proteins (Lötvall et al., 2014). Interestingly, PDEVs usually comprise a low concentration of proteins, including cytosolic proteins like actin and proteases, and membrane proteins acting as channels and transporters, like aquaporins and chloride channels (Zhang et al., 2016a; Alzahrani et al., 2023; Ipinmoroti et al., 2023; Wang et al., 2023). Cao and others detected 3,129 proteins in PDEVs derived from *G. quinquefolium* by mass spectrometry. According to Gene Ontology, these proteins were categorized into three groups, which are biological processes, cellular compartments, and molecular functions (Cao et al., 2019; Ou et al., 2023). Other groups analyzed the protein composition of PDEVs derived from *Citrus limon* and identified 162 proteins related to transportation (Stanly et al., 2019). Surprisingly, 56.7% of these proteins matched with proteins extracted from MEVs, according to the ExoCarta protein database (Fang and Liu, 2022b). However, extracellular vesicles derived from mammals comprise over 1,000 proteins, whereas PDEVs, like those obtained from *G. quinquefolium*, only have 28 proteins (Ju et al., 2013; Zhang et al., 2016a). Interestingly, some studies reported that the surface protein of PDEVs derived from *Allium sativum* may control their absorption by HepG2 (Song et al., 2020). Subsequently, they found a significant decrease in the absorption of PDEVs after inhibiting CD98 receptors on HepG2. In detail, Song et al. indicated that the interaction of lectin-like protein from PDEVs with the glycoprotein CD98 on the cell surface facilitated the uptake of *A. sativum*-PDEVs. This discovery provided a deeper comprehension of how cells take up PDEVs. On the other hand, the PDEVs contain proteins that help the plant to be resistant to stress (Rutter and Innes, 2017). For example, Rutter et al. detected the proteomics of the apoplastic PDEVs from *Arabidopsis* leaves and revealed that they contained a high concentration of stress response-related proteins (De Vallée et al., 2023). In a recent study, researchers compared the proteomics between apoplastic and whole-leaf PDEVs from *A. thaliana*. As expected, PDEVs obtained through tissue rupture methods contained more composition of proteins than those obtained exclusively from extracellular fluid (Liu Y. et al., 2020). Furthermore, several proteins present in MEVs have also been identified in PDEVs, including GPI-anchored proteins, annexins, heat-shock protein 70 (HSP70), and aquaporin proteins (Ju et al., 2013). In summary, complete profiling of proteins inside PDEVs is quite tricky due to the varying protein databases needed for matching in different plants.

#### 3.2.3 Lipids

Lipids have been considered the main composition of PDEVs, as the lipid bilayer composition influences their efficiency taken up by recipient cells and used for therapeutic purposes. Moreover, the proportion of lipids has a crucial role in the drug encapsulation and transportation capacity of PDEVs. The diversity in lipid composition determines the physical distribution and transport function of PDEVs. For instance, the primary lipid in PDEVs of *Citrus paradisi* and *A. sativum* was phosphatidylcholine (PC). In contrast, PDEVs derived from *Z. officinale* mainly contained phosphatidic acid (PA) as their main lipid component (Wang et al., 2014; Teng et al., 2018). However, PDEVs have been found to lack cholesterol and sphingomyelin, unlike MEVs and synthetic liposomes (Teng et al., 2018). In addition, this peculiarity in lipid compositions impacted the distribution of PDEVs. For instance, the lipid composition and structure of PDEVs played a crucial role in their distinctive absorption by the cells in the intestine. PDEVs derived from *Vitis amurensis* were plentiful in PA (53.2%), primarily found in the intestinal tract, and rarely absorbed by intestinal stem cells and macrophages (Ju et al., 2013; Mu et al., 2014; Zhuang et al., 2015; Zhang et al., 2016a). By contrast, the enrichment of PC lipids by PDEVs determined the migration from the gut to the liver (Teng et al., 2018). The presence of phosphatidylcholine (PC) was observed to promote the movement of PDEVs into liver cells, as demonstrated by analyzing images of mouse intestinal cells and liver cells at 1–6 h after consuming fluorescence-labeled PDEVs (Teng et al., 2018). PDEVs derived from *Z. officinale* migrated from the gut to the liver via the bloodstream and were absorbed by hepatocytes and Kupffer cells, resulting in significant accumulation in the liver (Zhuang et al., 2015; Kilasoniya et al., 2023). In the same way, *Z. officinale*-PDEVs produced using the thin-layer dispersion technique were absorbed by RAW264.7 and colon-26 cells and persisted in the stomach, ileum, and colon, indicating a focus on the intestines (Zhang et al., 2017). Mouse intestinal stem cells rarely absorbed PDEVs derived from *V. amurensis*, whereas they could absorb recombinant liposomes, which then accumulated in the intestinal tract (Ju et al., 2013; Record, 2013). Therefore, it is crucial to lucubrate the lipid role of PDEVs to elucidate the mechanism of PDEVs and utilize them in drug delivery systems.

#### 3.2.4 miRNAs

miRNAs, which are small RNA molecules approximately 22 nt in size, do not possess coding properties. Their primary roles include regulating the cleavage or translation of mRNAs and inducing the expression of specific target genes (Bartel, 2004; Olmi et al., 2023). Numerous studies have confirmed the existence of abundant miRNAs in PDEVs, typically thought to regulate gene expression in mammals (Chin et al., 2016). Besides, PDEVs are also proposed to protect miRNAs against degradation during transportation. Some studies also implicated that PDEVs have the potential to facilitate the interaction between the gut microbiota and the host immune systems by impacting the composition of gut microbes due to their possession of diverse miRNAs (Teng et al., 2018). Previous studies have detected 418 miRNAs in different species of PDEVs, suggesting that the over-expressed miRNAs might participate in inflammatory responses and signaling pathways associated with cancer (Xiao et al., 2018). Moreover, various methods, including gel electrophoresis and mass spectrometry, have been applied to validate miRNAs in PDEVs derived from *V. amurensis* (Ju et al., 2013). For instance, Zhang et al. identified 125 different miRNAs in *Z. officinale*-PDEVs, mediating immunomodulatory or metabolic regulation (Zhang et al., 2016a; Yin et al., 2022). Recent studies have demonstrated the ability of miRNA from PDEVs to regulate the composition of the gut microbiota. For example, miR167a from *Z. officinale*-PDEVs may activate the aryl hydrocarbon receptor (AHR) signaling pathway to alleviate ulcerative colitis by promoting IL-22 expression (Teng et al., 2018). Zhou and others demonstrated that miRNA-2911 derived from *Lonicera sempervirens* has the ability to suppress Influenza Virus A (IVA) (Zhou et al., 2015). Hence, the miRNA derived from PDEVs holds promise for therapeutic implications. Accordingly, due to the ability of miRNA to engage in intercellular communication, further study of PDEVs may be considered as one of the most potential therapeutic strategies in the future, especially as drug delivery systems.

## 4 PDEVs-based bioactive molecule delivery systems for therapeutic agents

PDEVs contained compounds inherit the bioactive properties of their source plants, and at the same time, their distinct structures also suggest the potential in the field of biomolecule transportation. For example, PDEVs, consisting of a double layer of lipids, enable the incorporation of hydrophobic medications, enhance the effectiveness of medicines, and efficiently accommodate hydrophilic chemicals to prevent adverse effects resulting from drug leakage (Nemati et al., 2022). Recent studies have implicated that the application of PDEVs has been shown to enhance the bioavailability, solubility, and stability of biomolecules (Kalani et al., 2016). For example, the PDEVs derived from *V. amurensis* were applied to encapsulate the methotrexate (GDN-MTX), preserving the anti-inflammatory properties of GDN-MTX and enhancing the uptake of GDN-MTX by intestinal macrophages (Wang et al., 2014). Moreover, the GDN-MTX effectively alleviated symptoms of ulcerative colitis, reduced the secretion of pro-inflammatory cytokines by intestinal macrophages, and mitigated the adverse effects of MTX while playing a more substantial anti-inflammatory impact in the mouse colitis model (Wang et al., 2014). Recent studies also suggest that PDEVs from *V. amurensis* coated with white cell membranes on their surface have the ability to improve target and accumulate at the site of inflammation in various models. Furthermore, these membrane fusion techniques also were proven to improve the targeting of PDEVs, prolong the drug circulation time, and enhance the accumulation of the medicine in the target tissue (Wang et al., 2015). In addition, several researchers also found that combining PDEVs with ligands, such as folic acid (FA), could enhance their targeting ability. For example, FA-modified PDEVs derived from *Z. officinale* could encapsulate the doxorubicin without size change, delay the doxorubicin release, produce sustained cytotoxicity, and improve the tumor targeting ability (Zhang et al., 2016c). Similarly, functional heparin (HR)-modified PDEVs from *Citrus limon* could load doxorubicin with a slight size variation, which effectively improved the internalization efficiency of HR-PDEVs (Xiao et al., 2022). At the same time, the diverse endocytosis capacity of HR-PDEVs efficiently dissipated intracellular energy, decreasing drug efflux (Xiao et al., 2022).

**FIGURE 4:**
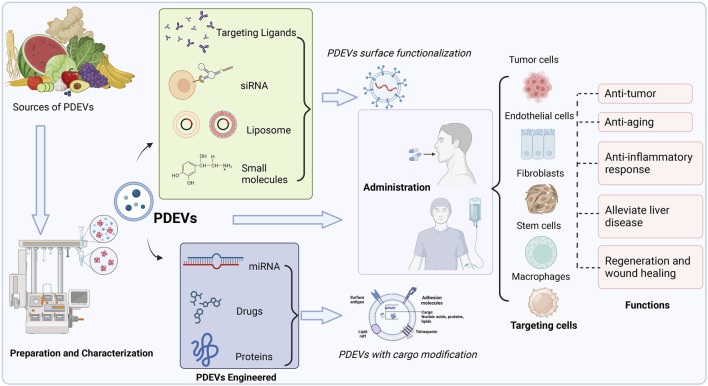
Multiple sources of PDEVs have been extensively used to develop novel drug delivery systems for treating particular diseases or maintaining normal functions. PDEVs and engineered PDEVs both are directly applied as therapeutic agents or delivery platforms, which could precisely target the different cells by administration to the body.

Accordingly, we summarized various PDEVs that have been extensively used to develop novel drug delivery systems for treating particular diseases or maintaining normal functions (Figure 4). Different edible and medical plants are applied to isolate therapeutic PDEVs with diverse functionalities. This review also lists the main types of PDEVs for their therapeutic applications (Table 3).

**TABLE 3:** Exploration, analysis, and potential medical applications of PDEVs.

Sources	Size (nm)	Drugs and contents	Target cells	Function	References
Grape	200–800 nm	proteins, lipids (PA, PE, PC), miRNA	Gastrointestinal tract (RAW-264.7)	Amelioration of DSS-induced colitis; Normalization of intestinal stem cell expression (Lgr5)	Ju et al. (2013)
Lemon	50–150 nm	Doxorubicin (DOX); miRNA, proteins, lipids	DOX-resistant ovarian tumor cells	Suppression of tumor cell growth and proliferation of chronic myeloid leukemia	Xiao et al. (2022)
Grapefruit	150–200 nm	Methotrexate (MTX), Doxorubicin (DOX), Curcumin (Cur)	Intestinal macrophages, Liver macrophages, Colon cancer	Enhances the chemotherapeutic inhibition of tumor growth through the delivery of therapeutic drugs	Wang et al. (2014)
	50–100 nm	miR17, miR-18a	GL26 brain tumor cells, Colon cancer	Amelioration in body weight loss and colon shortening in the mice model; Prolongation of survival rate	Zhuang et al. (2016)
	-	JSI-124, siRNA, paclitaxel (PTX)	Cancer cells	The enhanced therapeutical effect of IGNVs on inhibition of colitis; Prolongation of survival rate	Wang et al. (2013)
Broccoli	100 nm	AuNPs	Tumor cells	Amelioration of various types of colitis; Normalization of intestinal mucus layers	Deng et al. (2017), Singh et al. (2018)
Acerola	200–300 nm	miRNA	Bloodstream	Transports small RNA to the digestive system *in vivo*	Umezu et al. (2021)
Ginger	200–400 nm	Doxorubicin (Dox)	Colon cancer cells	Enhances the chemotherapeutic inhibition of tumor cell growth by delivering FA-mediated anti-cancer agents	Zhang et al. (2016c), Zhang et al. (2017)
			Colon-26 cells and HT-29 cells		
	100–200 nm	miRNA, proteins, lipids, siRNA-3WJ	Tumor cells (somatic cells, KB)	Inhibition of tumor cell growth with FA-3WJ siRNA ligand/GDENs	Li et al. (2018)
	50–100 nm	miRNA, proteins, lipids (PA, DGDG, MGDG, PC), metabolites (gingerol, shogaol)	Gastrointestinal tract (RAW 264.7)	Alleviation of the liver damage induced by alcohol	Zhuang et al. (2015)

### 4.1 PDEVs as inherent therapeutic agents

Despite being a relative innovation, PDEVs have already been employed to alleviate various diseases resulting from their advantageous healing properties and precise targeting of specific tissues (Wang et al., 2014). Especially, PDEVs were identified to bind to receptors and serve as recognition elements for remote locations due to the biomolecules composed in PDEVs, including proteins, lipids, and miRNAs (Wang et al., 2014; Teng et al., 2018). Therefore, these PDEVs may be directly applied as therapeutic agents. For instance, PDEVs from *V. amurensis* have been proven to relieve dextran sulfate sodium (DSS)-induced colitis in mice (Ju et al., 2013). Interestingly, these PDEVs also enhanced the growth of intestinal stem cells, which could potentially generate enduring intestinal organoid structures *in vitro*, possibly being the sole source of such stem cells (Deng et al., 2017). Furthermore, these PDEVs dramatically improve the regeneration of mucosal epithelium and successfully revive the structural integrity of the entire intestinal tract (Deng et al., 2017). Similarly, some studies found that PDEVs derived from *B. oleracea* displayed significant preventative and curative properties in relation to three forms of colitis (Wang et al., 2014). In detail, these PDEVs exhibit strong preventive properties against both acute and chronic colitis *in vivo* experiments. These were supported by evidence of heightened levels of anti-inflammatory cytokines and increased histoscore, as indicated by a decrease in colon shortening, loss of weight, and mucosa inflammation (Wang et al., 2014). Additionally, these *B. oleracea*-PDEVs were found to preserve the immune environment of the intestines while causing minimal negative responses by activating the Adenosine monophosphate-activated protein kinase (AMPK) pathway (Wang et al., 2014). Furthermore, PDEVs also have been reported to alleviate some liver diseases (Zhuang et al., 2015). *Zingiber officinale* has been applied in traditional medicine for centuries, providing numerous health advantages due to its inherent chemical components like shogaol and gingerol (Brahmbhatt et al., 2013; Kandemir et al., 2019). The expression level of a set of detoxifying/antioxidant genes, including glutamate-cysteine ligase modifier subunit (GCLM), heme oxygenase-1 (HO-1), glutamate-cysteine ligase catalytic subunit (GCLC), and NADPH quinone oxidoreductase-1 (NQO1), was increased by *Z. officinale*-derived PDEVs, leading to alleviating liver damage. In addition, the analysis of tissue showed that mice administered *Z. officinale*-PDEVs experienced a significant decrease in fat deposits within the liver. Moreover, *Z. officinale*-PDEVs markedly reduced levels of triglycerides and the weight of the liver. The mice that were given *Z. officinale*-PDEVs also exhibited histopathological alterations in the liver, indicating that PDEVs safeguard the liver against the toxicity of alcohol.

Interestingly, PDEVs also have dramatic anti-tumor activity. Raimondo et al. have reported that PDEVs derived from *Citrus limon* significantly suppressed the growth of tumors and reduced the multiple angiogenesis-related cytokines secretion in chronic myeloid leukemia (CML) through the Tumor necrosis-related apoptosis-inducing ligand (TRAIL) pathway (Raimondo et al., 2015). At the same time, these PDEVs targeted the tumor site within 15 min, exhibiting enduring effects lasting over 24 h *in vivo*. Actually, these PDEVs participate in cellular interaction and contribute to eliminating cancer cells via activation of TRAIL signaling, displaying their potential as chemotherapy agents (Raimondo et al., 2015). Similarly, PDEVs derived from citrus fruits have demonstrated the ability to suppress the survival of cancer cells, such as LAMA84, SW480, and A549, dependent on both the dosage and duration, compared to normal cell lines (HS5, HUVEC, and PBMC). Additional analysis indicated that these PDEVs induced apoptotic cell death in tumor cells by activating the TRAIL pathway, which was further validated in the xenograft model (Raimondo et al., 2018). Specifically, it was shown that the proteomics analysis revealed a correlation between the suppression of acetyl-CoA carboxylase (ACC) and the anti-cancer impact of these PDEVs, specifically in the case of colorectal cancer (Raimondo et al., 2018).

Numerous research studies have focused on the development of drug-delivery systems that possess the ability to target particular cells, tissues, or organs specifically. PDEVs exhibited a promising potential due to their inherent capacity to target desired tissues specifically. *Zingiber officinale*-PDEV exhibited distinct transport characteristics in a study focused on devising strategies to protect liver cells against the toxicity of alcohol. *Zingiber officinale*-PDEVs labeled with tetramethylindotricarbocyanine iodide (DiR) were observed to accumulate primarily in the liver *in vivo* assay. Using confocal microscopy to image immunostaining provided evidence that *Z. officinale*-PDEVs could enter and shift the hepatic vascular system. Caco-2 cells were found to internalize PDEVs derived from apples following co-incubation (Fujita et al., 2018). Therefore, the inherent targeting capabilities of PDEVs are expected to enhance their application as natural drug delivery systems.

### 4.2 PDEVs engineering

PDEVs have exhibited some inherent advantages, containing stability, firmness, and an appropriate structure, enabling the inclusion of drugs in their lipid bilayer and precise targeting of specific tissues (Wang et al., 2013; Wang et al., 2015; Teng et al., 2018). Recent studies also suggested that PDEVs could transport biomolecules safely and maintain long-term circulation in the blood when administered systemically. Besides inherent biological activities, PDEVs could be engineered as desired drug delivery platforms for the precise delivery of poorly soluble agents or therapeutic compounds, such as surface functionalization and content modification. Recent studies have focused on modification techniques to improve the drug delivery precision and efficiency of PDEVs (Chen et al., 2023).

#### 4.2.1 PDEVs membrane surface functionalization

Numerous studies have also indicated that PDEVs inherently possess the capability to target cells specifically. The surface of PDEVs, consisting of different lipids, exhibited distinct advantages like stability and precise targeting (Wang et al., 2013; Huang et al., 2023). The lack of studies compared with MEVs has resulted in limited knowledge about the fundamental mechanisms responsible for their natural targeting capabilities. However, recent studies implicated that the specificity of PDEVs is conferred by a specific alignment of proteins and lipids. According to research on the source plants, it has also been proposed that the selective absorption of PDEVs by particular cells is attributed to specific genes (that encode miRNAs and siRNAs) and small compounds, which act as external ligands for the targeting components on the cells (Wang et al., 2013; Teng et al., 2018). PDEVs exhibit excellent inherent targeting ability, minimal off-target effects, and rare toxicity compared to synthetic nanoparticles. It excludes the necessity for extra alterations to enhance biocompatibility, internal stability, and properties related to pharmacokinetics, which would otherwise be necessary to increase their effectiveness as treatments when they are modified with particular ligands. Furthermore, PDEVs are regarded as optimal nanocarriers due to their inclusion of typical proteins involved in transfer and trafficking (Ju et al., 2013; Wang et al., 2014). Although they possess inherent targeting characteristics, numerous endeavors have been made to refine their targeting effects and enhance the efficacy of their intracellular delivery (Teng et al., 2018). PDEVs could be developed to precisely and consistently target organs or cells by modifications using targeting ligands, like chemicals and genetic drugs on their membrane, and merging with cell membranes (Wang et al., 2013). These biomolecular modifications on the surface of PDEVs have proved to improve the stability and uniformity of the nanostructures (Li et al., 2018).

Several modification techniques focus on the surface functionalization of PDEVs to incorporate cell membranes or ligands. For instance, some studies focused on improving the precision of PDEVs, and the lipid-thin film is formed by adding small molecules like folic acid (FA) to the extracted lipids from PDEVs, which are applied to fabricate nanovesicles. It also demonstrated the targeting effect of the ligands exhibited on the membrane surface via the assay of biodistribution *in vitro* and *in vivo* (Wang et al., 2013; Yáñez-Mó et al., 2015). Following intravenous administration, some studies demonstrated that FA-PDEVs remained detectable even after 48 h of circulation *in vivo*, thereby enhancing their potential to infiltrate tumors. Thus, FA-PDEVs might serve as a perfect carrier for delivering drugs against tumors without causing the adverse impact of freely circulating medications. Some studies developed a drug delivery system utilizing *Z. officinale*-PDEVs combined with siRNA as targeting ligands (Adams, 1973; Lewis and Metcalf, 1988). Different physical configurations and angles, referred to as arrow-tail and arrow-head, were achieved by manipulating the RNA three-way junction (3WJ) architecture. The analysis of their absorption by cancer cells revealed varying degrees of cellular internalization based on the orientation of the 3WJ nanostructure. In order to selectively focus on cancer cells that have an overexpression of folate receptors, arrow-tail siRNA was conjugated with FA, which served as a targeting ligand and attached to the outer surface of the ginger-PDEVs. The decreased levels of survivin mRNA levels confirmed the therapeutic potential of FA-3WJ-PDEVs. These vectors led to a significantly enhanced distribution of tumor tissues compared to unmodified PDEVs (Wang et al., 2013). Recent studies also designed an intelligent delivery platform by utilizing PDEVs covered with a plasma membrane derived from activated leukocytes, aiming to improve their therapeutic capabilities and targeting precision (Wang et al., 2015). Moreover, using the extrusion technique, PDEVs were ready to create a personalized transport mechanism by merging with the cell membrane derived from leukocytes. Zhuang et al. also designed biomimetic nanoplatforms that integrated electrodynamic Pd-Pt nanosheets and PDEVs from *Z. officinale* (Qiao et al., 2022). These engineered PDEVs endow great anti-infection with prolonged blood circulation and accumulation at infection sites. In this process, the engineered PDEVs could specifically target inflamed tissues and diminish the off-side impacts while transporting drugs (Wang et al., 2015).

#### 4.2.2 PDEVs contents modification

The technology of encapsulation in the delivery system could transport biomolecules safely and maintain long-term circulation in the blood when administered systemically. As a result, PDEVs with content modification may be regarded as promising vectors for targeted delivery in tumorigenic disorders and chronic illnesses (Teng et al., 2018; Wang et al., 2019). Multiple techniques have been devised for loading diverse shipments into PDEVs. PDEVs can be loaded with desired cargo molecules using passive loading techniques, such as co-incubating with drugs (Vashisht et al., 2017). However, passive loading methods might not succeed in attaining a substantial encapsulation rate. Thus, subjecting PDEVs to sonication, freeze-thaw cycling, and similar mechanical treatments can destroy the integrity of their membranes and improve the efficiency of loading cargo. In order to prevent potential internal toxicity caused by impurities and achieve nanocarriers with consistent size, PDEVs can be designed using lipids and combined with cargo while preparing a lipid thin film (Li et al., 2018; Uddin et al., 2023). The method efficiently loads various shipments, including lipophilic drugs (DOX) and biomacromolecules like antibodies, siRNA, as well as DNA expression vectors (Wang et al., 2013; Zhang et al., 2016c). The encapsulation efficiency of doxorubicin through this method has resulted in high rates (95.9% ± 0.3%) (Zhang et al., 2016c). Moreover, conventional drug delivery systems loaded with some gene medicines, including siRNA and miRNA, have been lucubrated. However, the limited loading efficiencies of substances result in suboptimal therapeutic effects, and adverse consequences appear inevitable (Amreddy et al., 2017; Mainini and Eccles, 2020). In contrast, recent studies implicated that PDEVs could address these disadvantages (Zhu et al., 2022). For example, some studies suggested PDEVs from *Z. officinale* were used to carry specific siRNA targeting CD98 for the treatment of ulcerative colitis (Zhang et al., 2017). In comparison to a commercially accessible cholesterol and dioleoylphosphatidylethanolamine liposome product (DC-Chol/DOPE), it was identified that these PDEVs exhibited excellent biocompatibility, causing reduced toxicity and apoptosis in macrophage and eliminating colon-26 cells during *in vitro* assay (Zhang et al., 2017; Li et al., 2023). The PDEVs from *Z. officinale* were transfected with siRNA against CD98 using sonication to assess the mRNA expression of CD98. In detail, after being orally administered, the complexes of siRNA-CD98/PDEVs were successfully retained in the gastrointestinal tract and exhibited a notable decrease in CD98 expression in the gastrointestinal system compared to scrambled siRNA/PDEVs (Zhang et al., 2017). PDEVs derived from *Z. officinale*, which have low toxicity and high biocompatibility in contrast to synthetic liposomes, were discovered to be appropriate for transporting divalent metal-ion transporter-1 (Dmt1)-siRNA blunts to intestinal epithelial cells via reducing iron accumulation in hereditary hemochromatosis (Wang et al., 2019). Moreover, these PDEVs incorporating folic acid (FA) can enhance their capacity to specifically target the duodenum and seamlessly integrate into the intestinal tract through the proton-coupled folate transporter. In detail, they found that these PDEVs may decrease ferritin, transferrin saturation (TSAT), and non-heme Ferrum in multiple organs, such as the pancreas, heart, kidney, and liver, by reducing Dmt1-mRNA expression and iron loading (Wang et al., 2019).

### 4.3 Engineered PDEVs as the therapeutic system and bioactive molecule delivery system

Engineered PDEVs offer several unusual properties that render them highly appropriate as drug delivery systems for future medical purposes, such as transporting hydrophobic medications, improving the target precision, evading enzymatic decomposition, and eluding immune response. For example, some content modification of PDEVs has ensured excellent stability and biocompatibility in diverse physiological environments, including the bloodstream and different pH. All of these stellar features, coupled with the discovery of their intrinsic therapeutic actions, make engineered PDEVs desired for their advent into the arena of bioactive molecule delivery applications. Here, we shed light on engineered PDEVs as the therapeutic system and drug delivery systems.

Several studies have proved that the surface functionalization of PDEVs can prevent the harmful effects of chemotherapy by transporting a higher amount of medication to tumors rather than normal organs. For instance, FA-modified PDEVs are predominantly gathered in tumor tissues, whereas unmodified PDEVs are primarily localized to the spleen and liver. Interestingly, Zhang et al. also found that FA- PDEVs loading doxorubicin (DOX) has a dramatic effect on colon cancer (Zhang et al., 2016c). FA-PDEVs-DOX or FA-PDEVs were fabricated using a conventional approach that relies on the hydration of lipid films, which is similar to the technique employed for producing liposomes. Compared with unmodified PDEVs, FA-PDEVs showed an enhanced capacity to target colorectal tumor cells via active FA and folate receptor (FR) interactions. While the FA-PDEVs exhibited dramatic reduction levels within the spleen and liver compared to PDEVs. It suggests that utilizing FA-PDEVs as the delivery platform may diminish the systemic toxicity of drugs toward healthy tissues. Following intravenous administration, some studies demonstrated that FA-PDEVs remained detectable even after 48 h of circulation *in vivo*, thereby enhancing their potential to infiltrate tumors. Thus, FA-PDEVs might serve as a perfect carrier for delivering drugs against tumors without causing the adverse impact of freely circulating medications. In addition, recent studies also designed an intelligent delivery platform by utilizing PDEVs covered with a plasma membrane derived from activated leukocytes, aiming to improve their therapeutic capabilities and targeting precision (Wang et al., 2015). By modifying the membrane-coated PDEVs, specialized inflammatory receptor-enriched plasma membrane-coated PDEVs (IPDEVs) were obtained, which possess distinct cellular targeting capabilities. To evaluate the inflammatory potential of IPDEVs or PDEVs, they were introduced to the upper layer of a transwell plate containing a cultured monolayer of human umbilical vein endothelial cells (HUVECs), which served as a blood-brain barrier model. The study yielded more significant quantities of IPDEVs migrating through HUVECs within 48 h compared to PDEVs, and the inclusion of chemokines further improved the efficiency of migration. The impact of IPDEVs on peripheral circulation was further validated in models of inflammation and chronic inflammatory cancer models. Compared to PDEVs, the inflammatory sites exhibited a notable rise in the accumulation of IPDEVs, and *in vivo*, IPDEVs and DOX were consistently administered for tumor treatment. Compared to free-DOX and PDEVs-DOX, IPDEVs demonstrated an improvement in tumor permeability and a decrease in tumor volume. As the result of inflammation is a prevalent occurrence in numerous affected tissues, the exceptional capacity of these engineered PDEVs to specifically target sites of inflammation renders them highly effective nanocarriers.

In addition, the technology of encapsulation in the delivery system could transport biomolecules safely and maintain long-term circulation in the blood when administered systemically. Multiple studies suggested that PDEVs with content modification may be regarded as promising vectors for targeted delivery in tumorigenic disorders and chronic illnesses. As a result, they have the capability to transport medications with accurate localization to specific tissues instead of causing widespread effects throughout the body, resulting in enhanced therapeutic results and reduced side effects. To evaluate the drug-carrying capability of PDEVs, multiple groups assess the effectiveness of ginger-PDEVs in loading and releasing doxorubicin through a pre-loading strategy (Zhang et al., 2016c). Furthermore, the drug release kinetics of PDEVs containing doxorubicin and DC-Chol/DOPE liposomes loaded with doxorubicin were analyzed at different pH levels (pH 5.5, 6.0, 6.5, 7.0, and 7.5). PDEVs from *Z. officinale* carrying doxorubicin could effectively secret DOX in an acidic tumor microenvironment, and these PDEVs diffused more rapidly compared to the liposomes (Zhang et al., 2016c). The capacity of PDEVs derived from *Citrus paradisi* to offer precise drug delivery to intestinal macrophages was also assessed (Wang et al., 2013). According to their findings, the primary immune cells of the intestine, known as intestinal macrophages, took up the majority of PDEVs from *Citrus paradisi* in the lamina propria of both the small and large intestines (Wang et al., 2013). However, liposomes that are readily accessible in the market exhibited significantly lower efficacy in directing their action toward intestinal macrophages. Moreover, PDEVs from *Citrus paradisi* were developed to conjugate with methotrexate (MTX), a chemical drug that suppresses the immune response and reduces inflammation (Zhuang et al., 2016; Niu et al., 2021). In a mouse model of ulcerative colitis induced by DSS, PDEVs from *Citrus paradisi* loading MTX could alleviate the loss of body weight and the shortening of the colon. These findings indicate alleviated colon tissue damage and infiltration of inflammatory cells in mice treated with grape-PDEVs-MTX (Niu et al., 2021). Accordingly, the PDEVs-MTX were effective in targeting intestinal macrophages for treating inflammatory-related diseases in the intestines (Nishad et al., 2021; Niu et al., 2021).

## 5 Conclusion and future perspectives

Ever since the identification of PDEVs, they have demonstrated remarkable characteristics in the fields of biotherapy, delivering medication, and traversing physiological obstacles. PDEVs, derived from plant sources, are believed to hold immense promise in the field of biomedicine, particularly as drug delivery systems. PDEVs present excellent benefits compared to artificial carriers and MEVs in terms of drug encapsulation, resistance to gastrointestinal fluid, cellular absorption, efficiency, and safety. However, the technological limitations currently constrain the utilization of PDEVs as drug delivery systems.

Firstly, one of the primary challenges encountered by drug delivery applications of PDEVs is the absence of a universal and standardized approach to separation techniques. In the laboratory, various techniques like ultracentrifugation, density gradient centrifugation, ultrafiltration, and size exclusion chromatography find extensive application for the isolation and analysis of PDEVs. In comparison, the application of these techniques in the process of industrial transformation is hindered by the time-consuming and limited productivity. Researchers using ultracentrifugation may experience inconsistent results due to variations in centrifugation speed, force, and rotor type, as reported (Kameli et al., 2021b). Thus, it is imperative to investigate and develop a perfect isolation procedure to acquire consistent, steady, and productive PDEVs. Furthermore, it is also crucial to precisely analyze and regulate the quality and origin of raw materials owing to variations in plant types, growth phases, and geographical locations.

Further exploring the biological properties and transportation mechanisms of PDEVs remains one of the primary obstacles in utilizing them for drug delivery. PDEVs demonstrate exceptional tissue specificity and gastrointestinal resilience, yet this specific characteristic remains insufficiently elucidated. Further elucidating the tissue-specific targeting mechanism of PDEVs has the potential to overcome the challenge encountered in drug delivery applications. The ordered structure or lipid composition of PDEVs may enhance these capabilities of PDEVs. To understand these mechanisms of PDEVs, gaining a comprehensive comprehension of their physical characteristics might be the appropriate direction. The regulation of information in PDEVs appears to involve proteins, lipids, and miRNA, and the source of PDEVs may contribute to the variation in regulation. While articulating a comprehensive regulatory mechanism appears to be challenging, it may be more efficient to concentrate on regulating PDEVs in particular plants or species rather than across all plants. Simultaneously, the differentiation between regulatory mode and targeting mechanism facilitates the administration of diverse medications, enabling researchers to select from a range of PDEV origins based on their suitability for a particular ailment. Furthermore, the unpredictable element of the natural active component transported by PDEVs is not only capable of enhancing the effects of medications but also influencing their effectiveness (Nemati et al., 2022). Therefore, it is imperative to conduct comprehensive and profound research to elucidate the makeup of natural active constituents in PDEVs and the interaction mechanism between these constituents and medications to achieve efficient incorporation or collaborative therapeutic outcomes.

Insufficient targeting ability poses a challenge for the drug delivery application of PDEVs. On the other hand, proteins may have a critical role in receptor recognition. Several studies have indicated that PDEVs exhibit a lower protein count in comparison to MEVs, implying that PDEVs possess relatively limited selectivity (Pocsfalvi et al., 2018). The application of engineering was extensively utilized in the examination of MEVs, resulting in enhanced flexibility of MEVs (van der Koog et al., 2022). Thus, engineering PDEVs is expected to play a crucial role in the application of PDEVs as drug delivery platforms. PDEVs could also be modified using ligands like FA or peptides to enhance cargo targeting capability. The utilization of membrane fusion technology has the potential to combine PDEVs with synthesized liposomes, as well as mammalian EVs and bacterial EVs. The resulting hybrid PDEVs could potentially provide enhanced biological attributes. This strategy could improve the targeting precision and immune evasion capabilities of PDEVs.

In particular, several clinical studies on the therapeutic activities of PDEVs for clinical management have been registered (Alfieri et al., 2021). For example, the derivants of PDEVs as a delivery system to increase the bioavailability of oral curcumin are being tested on colorectal cancer patients (NCT01294072) (Wu et al., 2017). In a subsequent study, PDEVs derived from grapes and their derivates were proven to alleviate adverse reactions induced by chemoradiation in head and neck cancer patients, such as inflammatory and oral mucositis pain (NCT01668849) (Wu et al., 2017). Another clinical trial confirmed the prospects against inflammation of PDEVs from *Z. officinale* for effectively alleviating irritable bowel disease (NCT04879810) (Zhang et al., 2016a). Thus, considering these promising advantages, the researchers should carry out more preclinical and clinical trials to evaluate the bioactivities of PDEVs and engineered PDEVs in patients and define their minimum dosage. However, the application of PDEVs in clinical management is still limited by technology.

Firstly, one of the main problems of PDEVs translated from lab to clinical state is the lack of unified and standardized separation methods. These isolation methods have the problems of time-consuming and low yields in the industrial transformation (Kameli et al., 2021b). For example, ultracentrifugation may be a cost-effective and simple method, but it has been reported to disrupt PDEVs due to the centrifugation force and speed (Ramirez et al., 2018). Moreover, there have reportedly been inconsistencies in the separation of EVs by ultracentrifugation due to the rotor (Furi et al., 2017). Additionally, ultracentrifugation and other methods also have the risk of co-contamination with toxic proteins (Momen-Heravi et al., 2013). Further research of PDEVs to understand their biological role and transport mechanism in physiological or pathological processes may be one of the major challenges in the transformation from lab to clinical application (Cloet et al., 2021). For instance, components in PDEVs seem to be involved in the transport or regulation mechanism, and their different sources may be the primary reason (Kameli et al., 2021a). However, the current general regulation may be complicated to depict clearly. Therefore, it could be more effective to focus on the regulation of PDEVs in specific species rather than all plants.

Overall, drug delivery systems based on PDEVs demonstrated dramatic benefits in terms of biocompatibility, stability, distribution, and cellular absorption. Furthermore, employing PDEVs for the administration of biomolecules can decrease the adverse reactions of medications while also exhibiting the inherent capabilities of PDEVs. The unique attributes of PDEVs allow for the development of an innovative approach to drug delivery platforms. Since biomedical research on PDEVs is still in the infancy stages, there may be numerous challenges in utilizing PDEVs as drug carriers. Accordingly, further exploration of PDEVs in drug delivery is worthwhile, as these challenges can be overcome through additional research in the future.
